# Functional alignment maintains constant medial stability and a more distinct medial pivot pattern throughout flexion compared to mechanical alignment in medial pivot total knee arthroplasty

**DOI:** 10.1002/jeo2.70536

**Published:** 2025-11-14

**Authors:** Takashi Kobayashi, Kenichi Kono, Tomofumi Kage, Takaharu Yamazaki, Ryota Yamagami, Ryo Murakami, Takahiro Arakawa, Tomoki Murakami, Sora Koiwa, Kohei Kawaguchi, Shuji Taketomi, Hiroshi Inui, Tetsuya Tomita, Sakae Tanaka

**Affiliations:** ^1^ Department of Orthopaedic Surgery, Faculty of Medicine The University of Tokyo Tokyo Japan; ^2^ Department of Information Science, Faculty of Informatics Shonan Institute of Technology Fujisawa Japan; ^3^ Department of Orthopaedics, Saitama Medical Center Saitama Medical University Saitama Japan; ^4^ Department of Orthopaedic Biomaterial Science Osaka University Graduate School of Medicine Osaka Japan; ^5^ Master Course of Health Sciences, Graduate School of Health Sciences Morinomiya University of Medical Sciences Osaka Japan

**Keywords:** arthroplasty, functional alignment, kinematics, knee, replacement

## Abstract

**Purpose:**

To investigate whether functional alignment (FA) leads to differences in in vivo kinematics and early clinical outcomes compared to mechanical alignment (MA) in patients undergoing total knee arthroplasty (TKA) using a medial pivot (MP) prosthesis.

**Methods:**

Forty patients (20 MA and 20 FA) who underwent TKA with a MP implant were investigated under fluoroscopy during squatting using a two‐to‐three‐dimensional registration technique 1 year postoperatively. FA started with anatomical implant positioning plan, followed by navigation‐guided adjustments of femoral and tibial cutting plan based on intraoperative varus–valgus stress testing for soft tissue balance. In MA, resections are aimed for neutral coronal alignment. External rotation, varus–valgus angle, anteroposterior translation of medial and lateral contact points and kinematic pathways were assessed from 0° to 110° of flexion. Clinical outcomes were evaluated 1 year postoperatively using the Knee injury and Osteoarthritis Outcome Score (KOOS) and 2011 Knee Society Score (KSS). The range of motion and radiographic alignment were also evaluated.

**Results:**

A significant group × angle interaction was observed for lateral anteroposterior translation, external rotation and valgus angulation (*p* < 0.001), indicating different kinematic pathways between groups. In the MA group, a significant main effect of the flexion angle on valgus angulation was detected (*p* < 0.001), whereas in the FA group, the varus–valgus angles remained consistent throughout flexion. The FA group demonstrated significantly greater postoperative flexion; better KOOS scores for pain, ADL and sports; and superior KSS symptom scores (*p* < 0.05). Radiographic analysis showed significant differences in MPTA and LDFA in the FA group (both *p* < 0.001).

**Conclusion:**

FA and MA techniques resulted in distinct in vivo kinematics. The FA approach demonstrated MP knee motion with consistent varus–valgus stability throughout flexion, leading to improved early clinical outcomes compared to MA. These findings support the kinematic benefits of FA in MP TKA.

**Level of Evidence:**

Level III.

**Abbreviations**
2D/3D: two‐dimensional to three‐dimensional
ADL: activities of daily living subscale
ANOVA: analysis of variance
AP: anteroposterior
CPAK: Coronal Plane Alignment of the Knee
CT: computed tomography
FA: functional alignment
HKA: hip–knee–ankle angle
ICC: interclass correlation coefficient
KOOS: Knee injury and Osteoarthritis Outcome Score
KSS: 2011 Knee Society Score
LCS: local coordinate system
LDFA: lateral distal femoral angle
MA: mechanical alignment
MP: medial pivot
MPTA: medial proximal tibial angle
PROMs: patient‐reported outcomes measures
PS: posterior stabilised
PTS: posterior tibial slope
QOL: quality of life subscale
TKA: total knee arthroplasty

## INTRODUCTION

Medial pivot (MP) total knee arthroplasty (TKA) is designed to mimic the MP motion of the native knee by providing a highly constrained ball‐in‐socket structure of the medial compartment and flat surface in the lateral component [20, 36]. MP TKA has been reported to have higher anteroposterior (AP) stability compared to conventional posterior stabilised (PS) and posterior cruciate‐retaining designs, with reported satisfactory clinical outcomes [6, 19, 35, 41]. Additionally, MP TKA has been reported to exhibit kinematics closer to those of a healthy knee, as demonstrated in passive motion [9, 14, 37].

Functional alignment (FA) is an alternative alignment technique for TKA, in which alignment is adjusted by considering ligament balance using navigation or robotic assistance intraoperatively [7, 29]. FA TKA is predominantly performed using a computed tomography (CT)‐guided robotic arm [7, 17], although the application of other technologies, such as navigation systems is also increasing [11, 33]. In contrast to the mechanical alignment (MA) technique, conventionally performed to achieve neutral leg alignment by cutting the distal femoral and proximal tibial bones perpendicular to the mechanical axes, FA is a hybrid technique that begins with anatomical implant planning to reproduce constitutional coronal extension alignment and subsequently optimises implant positioning with ligament stress values throughout the surgical process, thereby achieving ideal kinematic characteristics precisely and reproducibly [5, 28, 32].

Accordingly, the FA method may be an appropriate soft tissue preservation technique to achieve a more physiological knee environment in MP TKA. The in vivo kinematics of MP TKA have been reported using the MA method [3, 37, 41]. However, no studies have evaluated the kinematics of MP TKA using the FA method, and the differences between the two techniques in the kinematics of MP TKA remain unknown.

Therefore, this study aims to clarify the differences in in vivo kinematics between MP TKA performed using the MA and FA techniques. The hypothesis of the study was that the two alignment methods would yield different kinematic patterns, with FA producing movements more closely resembling the native knee's pivotal motion compared to that of MA.

## METHODS

### Study design

This retrospective observational study compared consecutive series of MA and FA in MP TKA. A total of 40 knees that underwent MP TKA at our institution between December 2018 and January 2024 were included. All cases received a GMK Sphere (Medacta International S.A.) implant. Of these, 20 knees underwent the MA technique between December 2018 and December 2020, while 20 knees were operated using the FA technique between January 2021 and January 2024. MP TKA was initially performed using the MA technique at our institution, and patient satisfaction was favourable. However, the alignment technique (from MA to FA) was changed according to the period in seeking more satisfactory methods. The inclusion criteria for this study were as follows: (1) varus knee OA (hip–knee–ankle [HKA] angle <180°), (2) cruciate‐sacrificing TKA due to cruciate ligament dysfunction, (3) primary TKA and (4) consent for fluoroscopic evaluation. The exclusion criteria were as follows: (1) valgus knee OA (HKA angle >180°), (2) posttraumatic OA, (3) inflammatory arthritis (e.g., rheumatoid arthritis), (4) revision TKA, (5) no consent for fluoroscopic evaluation and (6) difficulty in deep squatting with a knee flexion angle of >110° during fluoroscopy.

### Surgical procedure

All surgeries were performed by the same surgical team using the GMK Sphere, cruciate‐sacrificing MP design implant and an image‐free navigation system (Precision N, Stryker Orthopaedics). Surgeries were performed using a medial parapatellar approach, and the patella was not everted. All components were implanted with cement.

In the MA group, the surgical approach was performed for all patients as previously reported [43]. Briefly, bone resection of the distal femur and proximal tibia were performed perpendicular to the mechanical axis of each bone to achieve an HKA of 0°. The femoral component was rotated parallel to the surgical transepicondylar axis and positioned to fit the anterior cortex without notching. The tibial component rotation was oriented parallel to Akagi's line [2], with a tibial posterior slope between 3° and 5°, according to the patient's anatomy.

In the FA group, surgery was performed according to the concept of FA [28, 29], beginning with a kinematically aligned osteotomy plan that accounted for an osteotomy depth equivalent to the implant thickness, based on a 2 mm thickness of unworn cartilage [1, 12, 25] and involved intraoperative adjustment of the bone‐cutting plan according to soft tissue balance. To achieve FA, valgus and varus stress was applied in both extension and 90° flexion to evaluate medial and lateral joint laxity during navigation. Based on joint laxity, the extent of femoral and tibial resection and the varus–valgus angle were adjusted as previously described [13]. This procedure was modified if the planned coronal resection angle was outside the safe zone of either a postoperative HKA (within ±3°) and/or independent femoral or tibial coronal cuts (within ±3°) [29], although limit of these restrictions may require further study [18, 22, 34, 42]. The protocols for femoral component fitting, tibial component rotation and tibial posterior slope were identical to those applied in the MA group. All patients began ambulation training the day after surgery, progressing through physical therapy that included range‐of‐motion exercises, gait training and subsequently stair climbing training.

### Kinematic analysis

The kinematic assessments were performed 1 year postoperatively as described previously [39]. Each patient was instructed to squat while using a single‐view fluoroscope in the sagittal plane. Consecutive movements were recorded using digital radiographic images with the same flat panel system, as previously reported [21]. The spatial position and orientation of the femoral and tibial components were evaluated using a two‐dimensional to three‐dimensional (2D/3D) registration techniques [44, 45]. The technique was performed using originally developed non‐commercial software (Kinematic Analysis) and is based on a contour‐based registration algorithm that utilises single‐view fluoroscopic images and 3D computer‐aided design models. The margin of error of the estimated relative motion between the metal components was ≤0.5° for rotation and ≤0.4° for translation [45]. In fixed‐bearing TKA, the insert can be considered rigidly connected to the tibial component, allowing the 3D position of the radiolucent polyethylene insert to be inferred from the estimated position of the tibial component. Based on the spatial position of TKA implants with tibial insert, the femorotibial contact point can then be reproduced [44]. The local coordinate system (LCS) for the components was established according to previously reported methods [39, 45].

The following kinematic parameters were evaluated for each knee flexion angle between 0° and 110°: femoral external rotation, defined as the axial rotation angle of the femoral component relative to the tibial component; femoral varus–valgus, defined as the varus–valgus angle of the femoral component relative to the tibial component; medial and lateral AP translation, indicates the AP translation of the medial and lateral femorotibial contact points; and kinematic pathways of the articular surface.

The knee flexion, femoral external rotation and varus–valgus angle were calculated according to a previously described method [10], and each angle of the femoral component relative to the tibial component was represented as a positive value. The anterior and posterior positions of the femoral component relative to the origin of the LCS of the tibial component were defined as positive and negative AP translation values, respectively.

### Radiographic evaluation

All patients were evaluated for lower limb alignment using CT scan preoperatively. The medial proximal tibial angle (MPTA) and lateral distal femoral angle (LDFA) were measured by CT employing the ZedKnee system (LEXI), and the HKA was formed by the medial angle of the mechanical axis of the femur and tibia [4]. These alignment measurements are typically obtained using long leg radiographs [8, 18, 25]. However, for consistency with the CT‐based bone model used for kinematics analysis, the same CT data were used to measure alignment based on anatomic landmarks, as reported previously [4, 30]. The postoperative CT scans were obtained 2 weeks after the surgery, and the MPTA, LDFA, HKA and posterior tibial slope (PTS) were measured. To provide an overview of the knee phenotype in the treated patient group, a postoperative phenotype classification of the knee was performed using the Coronal Plane Alignment of the Knee (CPAK) classification from the alignment parameters, where arithmetic HKA and joint line obliquity were calculated as the sum and difference of MPTA and LDFA, respectively, as previously reported [23]. To assess the interobserver reproducibility of the radiographic evaluation, all measurements were performed twice by 2 readers on 10 randomly selected radiographs. Intraclass correlation coefficients (ICCs) were used to assess the reliability of all measurements.

### Clinical evaluation

Knee extension and flexion angles were measured using a goniometer preoperatively and 1 year postoperatively. The Knee injury and Osteoarthritis Outcome Score (KOOS) [31] and 2011 Knee Society Score (KSS) [38], including patient satisfaction, were evaluated as patient‐reported outcome measures (PROMs) at the same time points. A higher score indicates better knee condition in both the KOOS and KSS.

### Statistical analysis

A priori power analysis was conducted using G*Power (version 3.1.9.4, Heinrich Heine University) to estimate the required sample size for detecting a significant interaction effect in a repeated‐measures analysis of variance (ANOVA) design. Assuming a medium effect size (Cohen's *f* = 0.25), *α* error probability of 0.05, power of 0.80, 2 independent groups and 12 repeated measurements (from 0° to 110°), the required total sample size was estimated to be 14 participants (7 per group). This sample size yields an actual statistical power of 0.87, which exceeds the conventional threshold of 0.80. Statistical analyses were performed using EZR (Saitama Medical Center, Jichi Medical University), which is a modified version of R Commander (version 4.2.1), and a graphical user interface for R (The R Foundation for Statistical Computing). The Shapiro–Wilk test was used to assess the normality of the kinematic data. Data are expressed as mean and standard deviation. Mann–Whitney *U* tests were used to compare continuous parameters, whereas Fisher's exact tests were used to compare categorical variables between MA and FA TKAs. Repeated‐measures ANOVA was conducted to examine whether the relationship between knee flexion angle and kinematic parameters (i.e., axial rotation, varus–valgus angle and AP translation) differed depending on the alignment method (MA vs. FA). This ‘group × angle’ interaction analysis enabled us to detect whether kinematic changes across flexion angles followed different patterns between the two alignment groups. When a significant interaction was found, post hoc pairwise comparisons (Bonferroni‐corrected) were tested to identify the specific flexion angles at which significant differences between groups occurred. To compare the total amount of change in the kinematic parameters observed throughout the range of motion, the difference in each kinematic parameter between the 0° and 110° flexion positions was compared between the two groups. To examine whether differences exist in the pivot patterns shown in the kinematic pathway, the pivoting ratio was calculated from the medial and lateral AP translations within (−1, 1) for each task, as reported previously [3]. The inter‐rater repeatability of each radiographic measurement was calculated using ICC. *p* values < 0.05 were used to denote statistical significance.

## RESULTS

The demographic characteristics of the patients who underwent MA and FA TKA are shown in Table 1. No significant differences in the preoperative demographic data were observed between the MA and FA TKA groups.

**Table 1:** Patient demographic characteristics.

	MA group (*n* = 20)	FA group (*n* = 20)	*p* Value
Age (years)	76.8 ± 6.5	74.5 ± 8.2	0.46
Sex (male/female)	6/14	7/13	0.99
BMI (kg/m^2^)	26.4 ± 3.6	26.3 ± 3.3	0.94
Preoperative maximum extension (°)	7.7 ± 5.2	7.3 ± 4.6	0.94
Preoperative maximum flexion (°)	118.0 ± 8.8	119.3 ± 8.2	0.70
Preoperative HKA (°)	176.0 ± 4.5	177.5 ± 3.2	0.12
Preoperative MPTA (°)	84.9 ± 3.7	84.9 ± 3.2	0.56
Preoperative LDFA (°)	88.9 ± 2.1	87.4 ± 2.0	0.08
Preoperative KOOS			
KOOS pain	50.1 ± 15.6	48.2 ± 18.9	0.53
KOOS symptom	53.6 ± 16.5	51.8 ± 23.3	0.74
KOOS ADL	55.1 ± 11.8	62.9 ± 17.6	0.07
KOOS sports	18.0 ± 17.9	23.5 ± 22.0	0.46
KOOS QOL	25.6 ± 13.5	30.3 ± 16.5	0.42
Preoperative KSS			
KSS symptom	7.6 ± 3.5	8.4 ± 5.4	0.69
KSS satisfaction	12.8 ± 4.2	12.9 ± 5.8	0.65
KSS expectation	12.8 ± 2.0	13.5 ± 2.1	0.21
KSS functional activity	41.8 ± 15.7	50.2 ± 14.8	0.08

*Note*: Data are presented as means ± standard deviation.

Abbreviations: ADL, activities of daily living subscale; BMI, body mass index; FA, functional alignment; HKA, hip–knee–ankle angle; KOOS, Knee injury and Osteoarthritis Outcome Score; KSS, 2011 Knee Society Score; LDFA, lateral distal femoral angle; MA, mechanical alignment; MPTA, medial proximal tibial angle; QOL, quality of life subscale.

### Femoral external rotation angle

**Figure 1:**
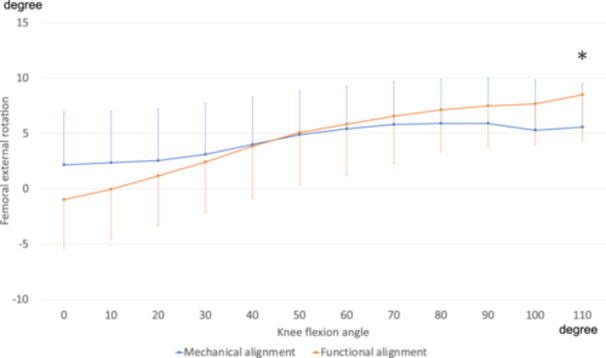
External femoral rotational angles. External rotation of the femoral component relative to the tibial component was defined as positive. *Significant group differences at individual angles are marked (*p* < 0.05, Bonferroni corrected). A significant group × angle interaction was observed (*p* < 0.001, repeated‐measures analysis of variance [ANOVA]).

Figure 1 shows the femoral external rotation angles. External rotation was significantly greater in the FA group (9.0° ± 4.0) at flexion angle 110° than in the MA group (5.6° ± 4.1, *p* = 0.04). Repeated‐measures ANOVA revealed a significant group × angle interaction between the groups (*p* < 0.001), suggesting a significantly different pattern of external rotation with flexion in the FA group. Additionally, the amount of change in the range of motion from 0° to 110° was larger in the FA group (9.8° ± 3.1) than in the MA group (5.5° ± 2.7, *p* < 0.001) (Table 2).

**Table 2:** Comparison of kinematic parameter changes in the range of motion from 0° to 110° between the groups.

	MA group (*n* = 20)	FA group (*n* = 20)	*p* Value
Medial AP translation (%)	−6.7 ± 3.1	−5.2 ± 2.5	0.13
Lateral AP translation (%)	−14.2 ± 6.3	−21.3 ± 6.2	**0.001**
Femoral external rotation (°)	5.5 ± 2.7	9.8 ± 3.1	**<0.001**
Femoral valgus angle (°)	1.2 ± 1.7	0.1 ± 1.7	0.10
Pivoting ratio	0.34	0.61	**<0.001**

*Note*: Pivoting ratio was calculated as (lateral AP translation – medial AP translation)/(lateral AP translation + medial AP translation), where values range from −1 to 1. Positive values indicate medial pivoting as described by Alesi et al. [3]. Data are presented as means ± standard deviation. The bold type means significant.

Abbreviations: AP, anteroposterior; FA, functional alignment; MA, mechanical alignment.

### Femoral varus–valgus angle

**Figure 2:**
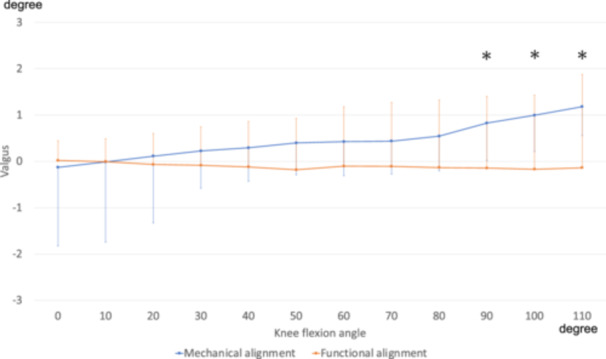
Femoral varus–valgus angles. The valgus angle of the femoral component relative to that of the tibial component was defined as positive. *Significant differences between groups (*p* < 0.05). A significant group × angle interaction was observed (*p* < 0.001).

The femoral varus–valgus angles were significantly greater in the MA group at high flexion angles of 90°, 100° and 110° (*p* = 0.03, 0.02 and 0.05, respectively) than in the FA group. A significant group × angle interaction was observed (*p* < 0.001), indicating that the pattern of valgus changes differed between the two groups (Figure 2). In the MA group, a trend toward a main effect of the flexion angle on valgus alignment (*p* = 0.06) was noted, suggesting that the valgus angle varied with knee flexion. In contrast, in the FA group, no significant main effect of the flexion angle was observed (*p* = 0.94), indicating that the varus angle remained consistent throughout the flexion range. These results suggest that the FA group exhibited a more stable varus–valgus alignment across the full range of motion, whereas the MA group demonstrated angle‐dependent valgus changes, contributing to a significant group × angle interaction.

### Medial and lateral AP translation

**Figure 3:**
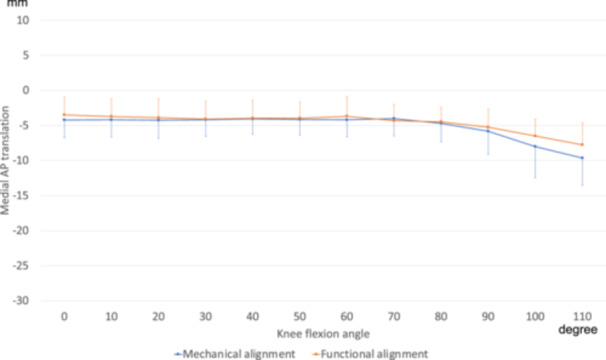
Anteroposterior (AP) translation of the medial contact point. The anterior translation of the femoral component relative to the tibial component was defined as positive. No significant differences were found between the groups at any individual flexion angle (*p* > 0.05), and no significant group × angle interaction was observed (*p* = 0.22).

For medial AP translation, no significant differences were found at any individual flexion angle (*p* > 0.05), and no significant group × angle interaction was observed (*p* = 0.213) (Figure 3). The amount of translation in the range of motion from 0° to 110° was also similar between the groups, as shown in Table 2.

**Figure 4:**
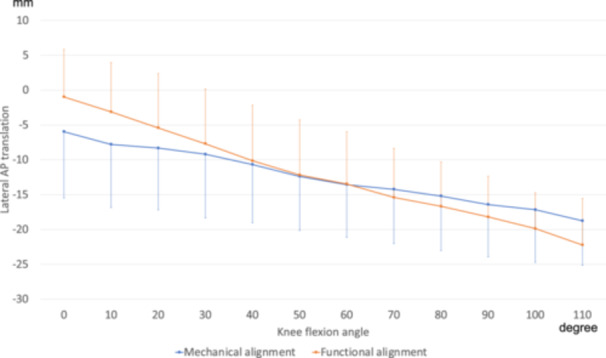
Anteroposterior (AP) translation of the lateral contact point. The anterior translation of the femoral component relative to the tibial component was defined as positive. Although no significant differences were found between the groups at any individual flexion angle, a significant group × angle interaction was observed (*p* < 0.001).

For the lateral AP translation, although no significant differences were found at any individual flexion angle (*p* > 0.05), a significant group × angle interaction was found between the groups (*p* < 0.001), suggesting a significantly different pattern of lateral AP translation with flexion in the FA group (Figure 4). Moreover, the amount of change in the range of motion from 0° to 110° was larger in the FA group (−21.3% ± 6.2) than in the MA group (−14.2% ± 6.3, *p* = 0.001).

### Kinematics pathway

**Figure 5:**
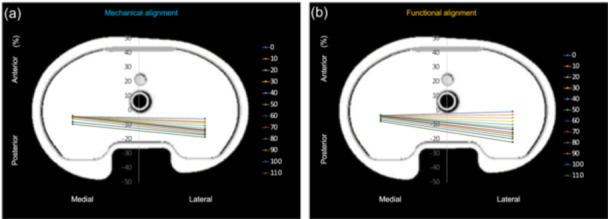
Kinematic pathways of joint surfaces in the (a) MA and (b) FA groups. The pivot ratio was significantly higher in the FA group than in the MA (0.61 vs. 0.34, *p* < 0.001). FA, functional alignment; MA, mechanical alignment.

Figure 5 shows the kinematic pathways of the two groups with MP patterns. The FA group showed a higher pivoting ratio (0.61) than the MA group, suggesting a more MP pattern (0.34, *p* < 0.001).

### Radiographic and clinical outcomes

**Figure 6:**
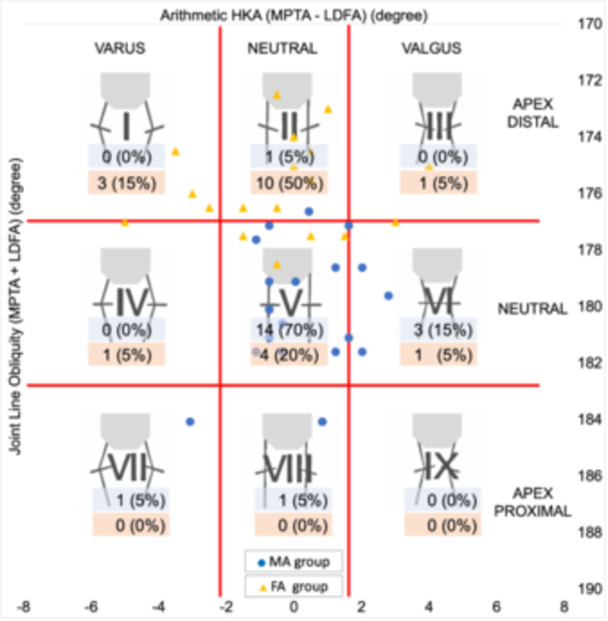
Overview of the knee phenotypes of the postoperative population included in this study. Plot of arithmetic HKA against calculated joint line obliquity for the postoperative population, showing distribution of the numbers and percentage of the nine CPAK phenotypes. The blue and orange colours indicate the MA and FA groups, respectively. The arithmetic HKA and joint line obliquity were calculated by the difference and sum of MPTA and LDFA as reported by MacDessi et al.'s [23] CPAK classification. CPAK, Coronal Plane Alignment of the Knee; FA, functional alignment; HKA, hip–knee–ankle angle; LDFA, lateral distal femoral angle; MA, mechanical alignment; MPTA, medial proximal tibial angle.

An overview of the knee phenotypes of the postoperative population included in this study is demonstrated in Figure 6. The most common phenotype of CPAK classification were Type V (14 knees, 70%) in the MA and Type II (10 knees, 50%) in the FA group. Table 3 shows the postoperative lower limb alignment of the two groups. The MPTA and LDFA were significantly different between the groups. ICCs for interobserver reproducibility were 0.92–0.97 for all alignment measurements. The postoperative clinical outcomes are summarised in Table 4. The FA group showed significantly greater maximum flexion (121.8° ± 7.3) compared to the MA group (116.3° ± 8.3, *p* = 0.03). Among KOOS subscales, the FA group demonstrated significantly higher scores in pain (93.6 ± 7.0 vs. 87.1 ± 9.3, *p* = 0.02), activities of daily living (ADL) (89.1 ± 11.8 vs. 85.1 ± 7.8, *p* = 0.03) and sports (64.8 ± 29.3 vs. 44.8 ± 24.4, *p* = 0.02). For 2011 KSS, the symptom subscale was also significantly higher in the FA group than in the MA group (22.4 ± 2.7 vs. 19.9 ± 3.9, *p* = 0.04). No significant differences were observed in other KOOS or KSS domains.

**Table 3:** Postoperative radiographic findings.

	MA group (*n* = 20)	FA group (*n* = 20)	*p* Value
Postoperative HKA (°)	180.2 ± 1.8	179.5 ± 2.1	0.40
Postoperative MPTA (°)	90.1 ± 1.2	87.7 ± 1.2	**<0.001**
Postoperative LDFA (°)	89.9 ± 1.5	88.1 ± 1.4	**<0.001**
Postoperative PTS (°)	3.0 ± 0.9	3.9 ± 1.4	0.06

*Note*: Data are presented as means ± standard deviation. The bold type means significant.

Abbreviations: FA, functional alignment; HKA, hip–knee–ankle angle; LDFA, lateral distal femoral angle; MA, mechanical alignment; MPTA, medial proximal tibial angle; PTS, posterior tibial slope.

**Table 4:** Postoperative range of motion, KOOS and KSS scores.

	MA group (*n* = 20)	FA group (*n* = 20)	*p* Value
Postoperative maximum extension (°)	2.5 ± 2.8	2.7 ± 2.4	0.59
Postoperative maximum flexion (°)	116.3 ± 8.3	121.8 ± 7.3	**0.03**
KOOS pain	87.1 ± 9.3	93.6 ± 7.0	**0.02**
KOOS symptom	84.0 ± 14.7	88.9 ± 13.8	0.19
KOOS ADL	85.1 ± 7.8	89.1 ± 11.8	**0.03**
KOOS Sports	44.8 ± 24.4	64.8 ± 29.3	**0.02**
KOOS QOL	64.2 ± 22.4	68.8 ± 19.7	0.56
KSS symptom	19.9 ± 3.9	22.4 ± 2.7	**0.04**
KSS satisfaction	27.7 ± 6.7	31.6 ± 6.2	0.10
KSS expectation	9.9 ± 2.6	10.5 ± 2.6	0.51
KSS functional activity	70.5 ± 17.4	76.0 ± 17.2	0.33

*Note*: Data are presented as means ± standard deviation. The bold type means significant.

Abbreviations: ADL, activities of daily living subscale; FA, functional alignment; KOOS, Knee injury and Osteoarthritis Outcome Score; KSS, 2011 Knee Society Score; MA, mechanical alignment; QOL, quality of life subscale.

## DISCUSSION

The most important finding of the present study was that the FA group exhibited a significantly more MP pattern than that in the MA group, characterised by comparable medial AP and varus–valgus stability, but with larger lateral rollback and external rotation pattern. The FA group functioned in vivo in a manner similar to that of the native knee as previously reported [9, 14]. In addition, despite the short‐term results, the FA group showed better flexion range of motion and favourable clinical outcomes in the KOOS subscales for pain, ADL and sports, and in the 2011 KSS symptom subscale than did the MA group. FA TKA is often performed using a CT‐guided robotic arm; however, successful outcomes have been reported with other technologies, and the favourable functional results of the present study corroborate those findings [11, 24].

Although the report did not compare kinematic differences after MP TKA using the MA and FA methods, several studies support the results regarding the relationship between kinematics and clinical outcomes. Alesi et al. [3] performed a radiostereometric analysis of 18 MP TKA using the MA technique, evaluating sit‐to‐stand and lunge motion and analysing the relationship between AP translation between the femur and tibia and clinical outcomes. Although this study was limited to the MA method, the group with more MP exhibited better clinical outcomes. Shimmin et al. [40] performed a perspective kinematic analysis of the pivoting, kneeling, running and stair climbing movements in 18 cases of MP TKA using the MA technique. They reported that the medial AP movement was stable, the lateral showed MP movement with an average of 7 mm of posterior movement, and no paradoxical forward movement of the femur was observed in any of the movements, which were consistent with normal knee kinematics patterns. Mizu‐Uchi et al. [27] analysed the stair‐climbing behaviour of 78 patients who underwent PS TKA using fluoroscopic analysis and reported that the 2011 KSS score was better in patients with a more MP pattern.

Kaneda et al. [16] compared rotational kinematics between alignment methods by analysing stair climbing and lunge movements in 10 kinematically aligned and 10 MA TKAs with MP implants, using a 2D/3D registration method without clinical outcomes. The study reported that kinematically aligned TKA showed a better MP pattern than MA TKA by showing a greater lateral posterior translation with minimal medial AP translation. Based on the intraoperative gap balance perspective, greater tibial internal rotation and lateral laxity were observed during knee flexion in kinematically aligned compared to MA TKA [26]. Similarly, Kaneda et al. highlighted that in the context of an MP knee prosthesis, a highly congruent medial compartment and less‐conforming lateral compartment may easily reproduce an MP motion closer to that of the native knee, and kinematically aligned techniques further enhanced this MP pattern and femoral external rotation relative to the tibia [16].

In the MA group, the valgus angle increased depending on the angle of knee flexion (1.2° ± 1.7 valgus from 0° to 110° flexion), whereas in the FA group, the angle of varus–valgus remained constant throughout the entire range of flexion (0.04° varus on average) (Figure 2). To the best of our knowledge, only 1 report described the differences in the in vivo kinematics of varus/valgus angles after TKA based on alignment techniques between MA and FA methods [15]. They compared the in vivo kinematics of bicruciate‐retaining TKA performed using the MA and FA methods and reported that the MA method tended to show a valgus alignment compared to FA TKA overall. Matsumoto et al. [26] reported intraoperative soft tissue balance in modified kinematically TKA and MA TKA, and highlighted that the varus/valgus ligament balance in patients with kinematically aligned group was significantly higher at high flexion angles of 90° and 120° than in those of the MA group. These results are consistent with those of this study. In contrast, Qordja et al. [30] investigated dynamic intraoperative HKA in robot‐assisted kinematically aligned TKA in the range of motion from 0° to 120°. The results showed that the HKA obtained in extension (176.1 ± 3.7°) revealed a varus trend of intraoperative HKA through mid‐flexion (175.1 ± 3.8°), followed by a decreasing varus trend through deep flexion (175.8 ± 4.2°). This result demonstrates a kinematics different from those in the FA group in the present study but requires careful interpretation, as the extension mechanism was not closed due to the presence of a robotic tracker pin in the joint, and the data were obtained in a passive motion under anaesthesia. The factors contributing to a stable varus–valgus balance in the FA method are considered to reflect the advantages of the FA technique, which involves adjusting the cutting angle and amount after evaluating joint laxity by applying varus–valgus stress [13]. In the MA method, femoral posterior condylar osteotomy is performed in an externally rotated position relative to the posterior condylar axis (parallel to the surgical epicondylar axis), which is thought to induce increased valgus during deep flexion [29].

This study had some limitations. First, MA TKA was performed prior to FA TKA; therefore, the FA group may have benefited from a longer learning curve in performing MP implantation, which could have influenced the results or introduced bias. Second, the sample size was small. Although a priori power analysis indicated that a total sample size of 14 (7 per group) would be sufficient to detect medium‐sized group × time interactions (*f* = 0.25) with 80% power and 12 repeated measurements, the actual sample size (20 per group) provides more than adequate power overall. However, given that the between‐group effect sizes varied across flexion angles, the statistical power to detect differences may have been limited at specific angles (i.e., from 20° to 80°) where the effect size was relatively small. Consequently, the current sample size (20 per group) may have been underpowered to detect statistically significant differences at certain flexion angles where the effect size was smaller, and the clinical relevance of these findings should be interpreted with caution. Moreover, the number of patients who agreed to undergo fluoroscopy was limited due to concerns regarding radiation exposure. Patients unable to achieve knee flexion beyond 110° during fluoroscopy due to insufficient muscle strength or range of motion were excluded, further restricting the sample size and introducing a major selection bias over the relatively long study period. Third, only postoperative in vivo kinematics were assessed in the present study. Preoperative in vivo kinematics may affect postoperative kinematics. Fourth, only squatting kinematics were evaluated. The results may differ for different activities such as stair stepping, kneeling or walking. Additionally, the target value of PTS was not fixed between 3° and 5° in the present study, suggesting that variations in PTS could potentially influence AP kinematics and femoral rotation. However, in this study, which employed MP TKA with high medial constraint, the effect of PTS was considered negligible, as the correlation coefficient of these kinematics was markedly low (*r* = −0.003 to 0.21) and PTS did not differ between the two groups. In addition, although no failures occurred, such as evident loosening or revision in the patient group, long‐term survival and complications remain unknown. Nevertheless, to the best of our knowledge, this is the first report to compare the differences in in vivo kinematics and clinical outcomes after MP TKA performed using the MA and FA methods, highlighting its clinical relevance.

## CONCLUSIONS

During squatting, FA in MP TKA demonstrated greater lateral posterior translation and external rotation, while maintaining medial AP and varus–valgus stability, resulting in a more pronounced MP pattern compared to MA. Moreover, FA was associated with improved short‐term clinical outcomes, suggesting potential for broader application in the future.

## AUTHOR CONTRIBUTIONS

Takashi Kobayashi performed the study, formal analysis and wrote the manuscript. Kenichi Kono supervised the research and conceived the study design. Tomofumi Kage, Tomoki Murakami and Sora Koiwa assisted with the data collection. Takaharu Yamazaki, Ryota Yamagami, Ryo Murakami and Takahiro Arakawa provided technical assistance. Tetsuya Tomita, Kohei Kawaguchi, Hiroshi Inui, Shuji Taketomi and Sakae Tanaka provided general support. All authors read and approved the final manuscript.

## CONFLICT OF INTEREST STATEMENT

The authors declare no conflicts of interest.

## ETHICS STATEMENT

Approval was granted by the University of Tokyo Institutional Ethics Review Board (number 10462‐[2]). Informed consent was obtained from all participants included in the study. Moreover, the patients signed informed consent forms for the publication of their data and photographs.
